# Perceptions and attitudes toward palliative care among healthcare professionals in Qatar’s home care setting

**DOI:** 10.3389/fmed.2025.1678462

**Published:** 2025-10-09

**Authors:** Feras Haddad, Gary E. Day, Sybil George, Brijesh Sathian, Hanadi Al Hamad, Essa Al-Sulaiti

**Affiliations:** 1 Home Healthcare Service, Hamad Medical Corporation, Doha, Qatar; 2 ECA College of Health Sciences, Brisbane, QLD, Australia; 3 Department of Geriatrics and Long-Term Care, Rumailah Hospital, Doha, Qatar

**Keywords:** palliative care, end-of-life, Qatar, home healthcare, attitudes, barriers

## Abstract

**Introduction:**

Palliative care (PC) is an emerging concept in the Middle East, with Qatar lacking formalized home-based services until the 2021 Doha declaration. This qualitative study addresses a gap in the literature by exploring home healthcare service (HHCS) staff perceptions, attitudes, and challenges in delivering PC in Qatar’s home setting, aiming to inform service development and enhance end-of-life care accessibility.

**Methods:**

Using Braun and Clarke’s thematic analysis approach, semi-structured interviews were conducted with 13 purposively sampled HHCS staff from various disciplines (e.g., physicians, nurses, pharmacists). Interviews, lasting approximately 30 min each, were audio-recorded via Microsoft Teams, transcribed, and analyzed with QualCoder software to identify codes, sub-themes, and major themes.

**Results:**

Four key themes emerged: (1) patient-centered care and comfort, emphasizing pain-free management, quality of life, and respecting patient/family wishes; (2) challenges and barriers, including narcotic drug access, community acceptance, and cultural/religious considerations; (3) communication and supportive services, highlighting clear channels and psychosocial/spiritual support; and (4) training and resources, stressing staff education, emotional support, and policy revisions.

**Discussion:**

Findings reveal unique Qatar-specific obstacles, such as regulatory restrictions on narcotics and cultural norms affecting death at home, while offering opportunities to improve training, policies, and awareness. These insights are crucial for integrating culturally sensitive PC into home-based systems, potentially reducing hospital burdens and aligning with global aging trends.

## 1 Introduction

In November 2021, the Doha Declaration on the Development of Palliative Care in Qatar was signed (1), paving the way for expanding palliative care (PC) services through a funded strategy and action plan, including the establishment of home-based PC services to improve accessibility and quality of care in Qatar. Prior to this, Qatar lacked formal systematic health care provision for patients with PC needs requiring home treatment by a specialized team, with PC limited to oncology patients in hospital settings. Qatar’s 5-year national health strategy outlines the vision for health sector growth (2), recognizing aging trends and increased life expectancy as challenges that heighten demand for services, including enhanced end-of-life (EoL) care. Globally, over 64 million people require PC annually, yet only about 14% receive it (3), with a recent study in Qatar indicating that 96% of patients were unaware of PC (4). Aligning with global aging patterns, Qatar’s population is projected to reach approximately 4.1 million by 2050 (5). In response, our existing Home Healthcare Service (HHCS) received business plan approval to establish a community-based PC service.

Palliative care remains a relatively new concept in the Middle East, first introduced in Saudi Arabia in 1992 and only recently expanding to countries like Qatar. Barriers to progress include resource shortages, limited education and awareness, and political challenges (6). Qatar’s first inpatient PC unit at Al Amal Hospital opened in 2008 with 10 beds for oncology patients (5); despite advance directives and Do Not Attempt Resuscitation (DNR) protocols since 2004, access remains suboptimal, with aggressive treatments, narcotic medication challenges, and most cancer deaths occurring in hospitals within one month of admission, while only 0.4% happen at home (5). Studies reveal training gaps: only 36.7% of medical oncologists and 31% of oncology nurses in Qatar have formal PC training, highlighting deficiencies in managing dying patients, pain, symptoms, and psychosocial, ethical, and spiritual issues (7, 8). Despite many patients wishing to die at home, most deaths occur in hospitals, overburdening healthcare system capacity, escalating costs, and diminishing patients’ quality of life (QoL), prompting calls for funded 24-h home-based PC (9). Additional research notes reduced treatment aggressiveness in EoL but underscores the absence of home PC to minimize hospital admissions and enhance QoL via better pain control and support (10). Pain management faces a culture of fear and regulatory barriers limiting narcotic use (11). As a nascent specialty in Qatar, research is essential to inform PC-related policies (12).

The reviewed literature on PC in Qatar focuses primarily on hospital and inpatient settings, revealing a gap in addressing homecare staff perceptions and attitudes. Hamad Medical Corporation (HMC) HHCS, the principal homecare provider, serves over 2,000 homebound adults most of whom are elderly, providing chronic disease management to enhance QoL (13).

This study seeks to deepen understanding of how healthcare professionals (HCPs) in Qatar’s homecare setting view PC delivery in the community, gathering insights from interviews with multidisciplinary HHCS staff to inform better strategies, frameworks, service design, and enhanced care for patients and families at home. The study aims to answer: How do healthcare professionals in Qatar’s home care setting perceive and approach palliative care, and what factors influence their attitudes and practices in providing it to patients?

The study has two main objectives:

1. To understand the perceptions and attitudes of HHCS staff in Qatar toward providing PC in the community.
2. To identify barriers or drivers to community PC provision, including health system barriers such as financing, training, policies, and narcotics access, recognizing practical, educational, cultural, or organizational factors supporting its implementation.

## 2 Materials and methods

### 2.1 Study design

The study used Braun and Clarke’s approach to thematic analysis (14) to gain in-depth insights into the participants’ perceptions and attitudes toward palliative care in the home care setting through semi-structured interviews. The study received academic and ethical approval from the University of Warwick under registration number: REGO-WMS-HL-24.008.

### 2.2 Recruitment and sample

The selection was based on purposive sampling, with an invitation email sent to 136 frontline staff members from various disciplines. Inclusion criteria were (1) Existing clinical staff employees for more than 12 months (2) Agreeing to attend an audio-recorded online interview with prior consent (3) English language proficiency. An information session was conducted, and 13 participants gave written consent to attend the interviews. The sample represented various clinical disciplines working in the home care service, including physicians (*n* = 3), nurses (*n* = 5), clinical pharmacists (*n* = 2), dieticians (*n* = 1), and respiratory therapists (*n* = 2). Although purposive sampling aimed to capture diverse perspectives, the small sample size may introduce selection bias, as participants were self-selected from those who responded to the invitation and met inclusion criteria. This limits the generalizability of the results to the wider population of healthcare professionals in Qatar’s home care settings. Data saturation was achieved, as no new themes emerged from the later interviews.

### 2.3 Interview process

The interviews were conducted with existing staff members in our homecare service. The interviews were performed online using the workplace Microsoft Teams application and were recorded using Microsoft Teams with integrated transcription, where each interview lasted 30 min on average. The interview consisted of four open-ended questions with prompts (see Appendix 1), focusing on key areas related to perceptions and attitudes toward PC.

### 2.4 Data analysis

**FIGURE 1:**
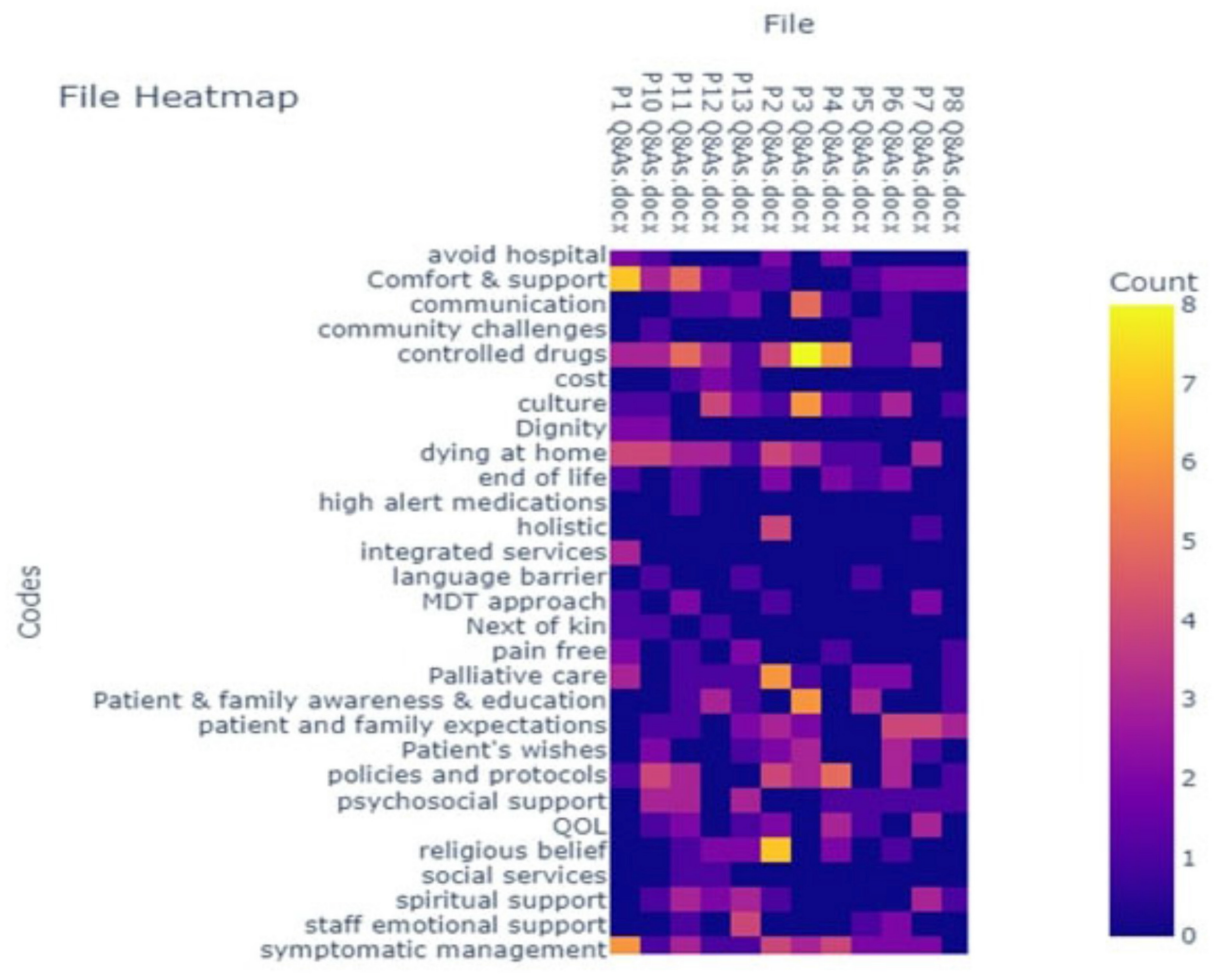
Codes heatmap.

**FIGURE 2:**
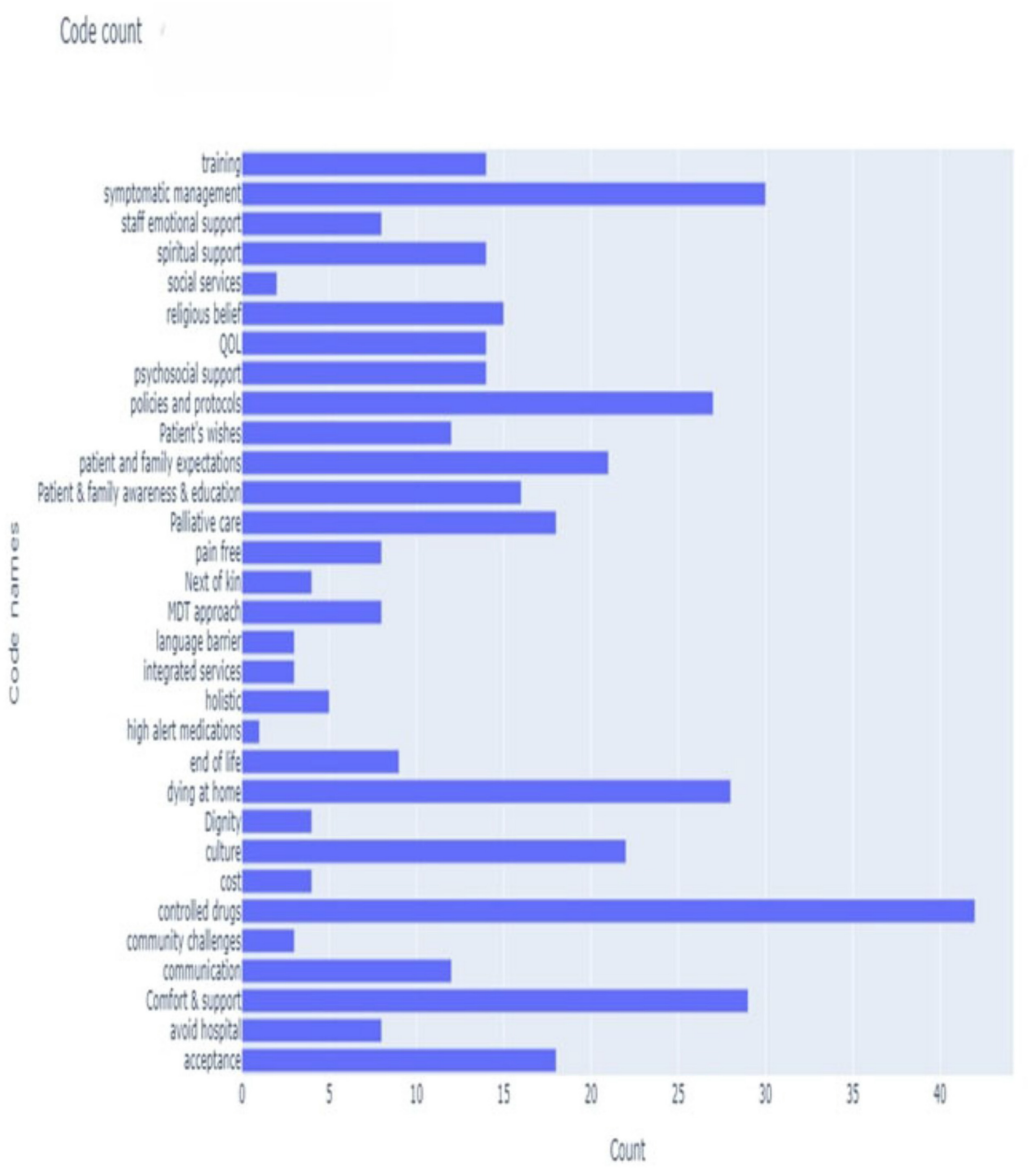
Bar chart: code count.

**FIGURE 3:**
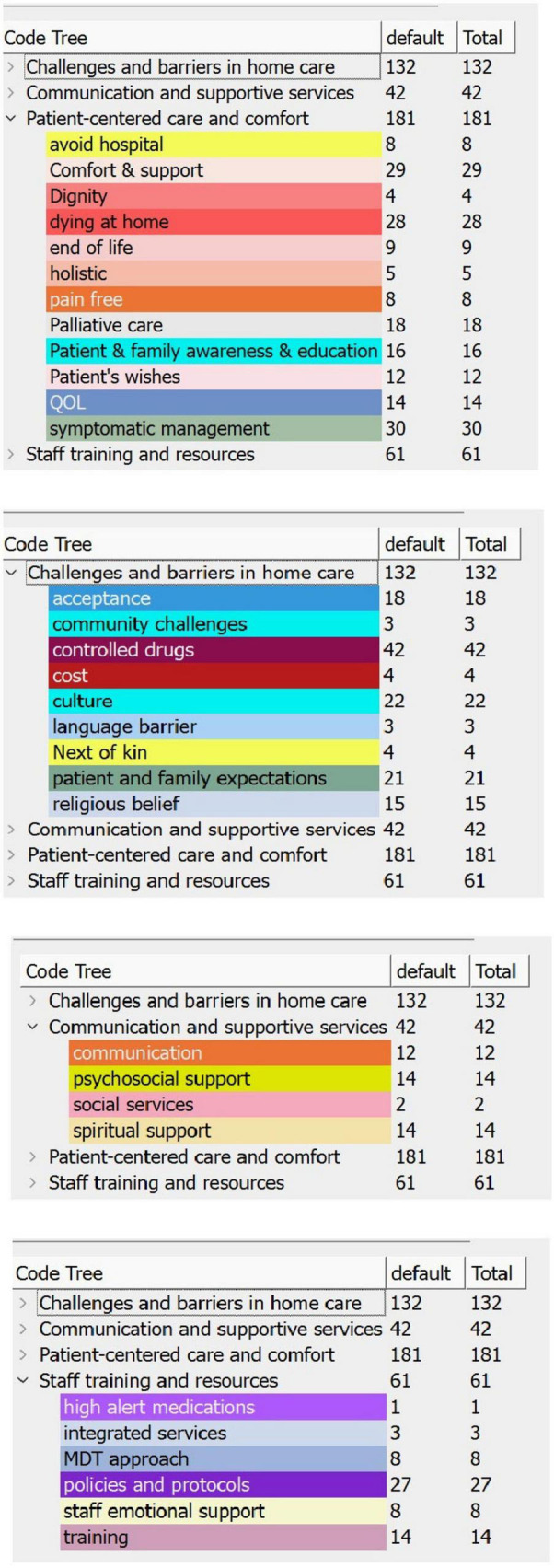
Code tree chart: counts of codes and categories.

This study used thematic analysis (14). The transcriptions of interview recordings were reviewed and then followed by the coding stage. The transcriptions were read and re-read to identify potential themes. Initial coding was done using QualCoder, a free open-source tool for qualitative data analysis (15). The answers from the participants to the interview questions informed the coding process and ensured retaining diversity. A code heatmap aided in identifying code frequency per participant (Figure 1). Codes were then reviewed based on the overall count across all participants (Figure 2). Codes were grouped into sub-themes and quotes were linked under each code and sub-theme. A further stage involved a further review of the codes creating categories (Figure 3) to define and name the main and final themes ready for results analysis (Table 1).

**TABLE 1:** Themes and sub-themes.

Theme	Sub-theme
1. Patient-centered care and comfort	1. Pain-free and comfort 2. Patient and family wishes and awareness 3. Death and dying at home
2. Challenges and barriers	1. Narcotic drugs 2. Acceptance and community challenges 3. Cultural and religious considerations
3. Communication and supportive services	1. Clear communication channels 2. Psychosocial and spiritual support
4. Training and resources	1. Staff training and emotional support 2. Policies and protocols

## 3 Results

### 3.1 Patient-centered care and comfort

The participants expressed a good understanding of PC and the difference between PC and EoL. More than half of the participants (*n* = 8) considered EoL to be the last 6–12 months of life. The main aim was to keep patients’ pain-free and comfortable to improve their QoL while respecting patient and family wishes in terms of managing symptoms and matters related to the preferred place of death.

a. Pain-free and comfort

All participants (*n* = 13) viewed their role in PC to provide support to terminally ill patients, ensuring their dignity and QoL are preserved, and they focused on the importance of alleviating their suffering by being pain-free.

When asked about the difference between curative and symptomatic management, some pointed out that PC isn’t about aggressive treatment that may not improve their QoL. Some believed pain was the main trigger for suffering and stress hence pain control should be a high objective in PC as illustrated by P1’s comment: “Keeping patients comfortable, pain-free, and dignified at the end of their life. Basically, to die comfortably.” Similarly, P9 emphasized, “To decrease the pain for the patient, that’s the main aim of this service” (Table 2).

**TABLE 2:** Quotes: pain-free and comfort.

Sub-theme	Quotes
Pain-free and comfort	*(P1): “Keeping patients comfortable, pain-free, and dignified at the end of their life. Basically, to die comfortably”*
	*(P2): “Sustaining treatments would not be suitable for them, would not add any more quality of life to their general status”*
	*(P4): “but rather than controlling the symptoms and improving the quality of life”*
	*(P9): “To decrease the pain for the patient, that’s the main aim of this service”*
	*(P4): “I just need the best for my patients. It will be being like pain-free and without any symptoms that may hinder his ability to perform most of his, well, daily living activities”*

b. Patient and family wishes and awareness

All participants (*n* = 13) acknowledged the importance of discussing patient and family wishes from the outset, to enable a holistic management approach considering those wishes to support setting realistic goals and plans of care. Patient and family awareness and education about PC and EoL were also frequently mentioned with P7 noting, “We should initially assess the patient’s preferences,” and P5 highlighting community misconceptions: “They need more awareness, more information to the families and patients in the community of Qatar, maybe it will also help. The awareness among our communities is not great, they think palliative care means the patient will die soon” (Table 3).

**TABLE 3:** Quotes: patient and family wishes and awareness.

Sub-theme	Quotes
Patient and family wishes and awareness	*(P7): “We should initially assess the patient’s preferences”*
	*(P10): “Because in the Community setting it will be very different than the hospital. So we need to understand what exactly the patient and the family need”*
	*(P13): “So I think as a medical staff, we have to understand the importance of listening. It isn’t that we don’t want to fight about the life of the patient or to live further. But sometimes the family is ready to let go”*
	*(P6): “I think we should respect that and let it be the way he wished for or she wished for”*
	*(P12): “Whoever is providing care at home, this is not just healthcare professional, but the caregivers and family members. Everybody should be educated properly because there is a lot of misconception about palliative care or the end-of-life care”*
	*(P5): “They need more awareness, more information to the families and patients in the community of Qatar, maybe it will also help. The awareness among our communities is not great, they think palliative care means the patient will die soon”*
	*(P3): “I think the knowledge level in the community and the understanding about what is palliative care is really not here in Qatar”*

c. Death and dying at home

Most participants (*n* = 11) felt that an effective PC service means avoiding hospital admission or death in hospital and allowing patients a peaceful death at home among their family members and loved ones if that was their preferred choice. An interesting view from one participant (P2) was that death in hospital would preserve the good memories the family had with the patient at home, as P10 described: “I feel that most of the patients will be comfortable at home as their favorite place to be with their family without a lot of suffering, and to have a peaceful death at home.” This preference was echoed by P4: “So my perspective is to provide care at home and try to minimize as much as possible ED and hospital admissions” (Table 4).

**TABLE 4:** Quotes: death and dying at home.

Sub-theme	Quotes
Death and dying at home	*(P1): “Palliative home care is a good way of keeping patients away from the hospital”*
	*(P4): “So my perspective is to provide care at home and try to minimize as much as possible ED and hospital admissions,” “The machines beeping around you and all those things are very stressful”*
	*(P10): “I feel that most of the patients will be comfortable at home as their favorite place to be with their family without a lot of suffering, and to have a peaceful death at home”*
	*(P2): “They wanted the home to be an environment where they only had good memories of the grandfather staying at home, playing with the children. They did not want that place to carry the memory of this person dying at home”*

### 3.2 Challenges and barriers

a. Narcotic drugs

All participants (*n* = 13) acknowledged the importance of timely access to narcotic medications to deliver effective pain management which is a key element in PC. However, they also voiced concerns about difficulties in accessing such medications, the injectable forms in particular, lack of protocols and pathways related to dispensing, storage in the home care hubs, transport, and administration. Furthermore, some thought it was something better left to be given in the hospital setting which meant the patient would have to be readmitted, especially when some families doubt its effect and view it negatively overall, exemplified by P2’s observation: “Controlled drugs are not something that we do in Qatar, but it’s widely practiced elsewhere in other countries. There are safe ways to administer drugs at home, and it’s an essential part of offering community-based palliative care.” P3 added concerns about legal and family perceptions: “I’ve never seen anyone at home on you know injections or anything like that and I think I don’t know if there’s some like legal issues about administering those in the home” (Table 5).

**TABLE 5:** Quotes: narcotic drugs.

Sub-theme	Quotes
Narcotic drugs	*(P2): “Controlled drugs are not something that we do in Qatar, but it’s widely practiced elsewhere in other countries. There are safe ways to administer drugs at home, and it’s an essential part of offering community-based palliative care. Without parenteral drugs end of life care in the community isn’t going to be feasible”*
	*(P11): “I don’t know how much of this is possible, especially the pain control medicines that we can give at home. It’s really difficult. If patients are in the hospital they will get more”*
	*(P3): “I’ve never seen anyone at home on you know injections or anything like that and I think I don’t know if there’s some like legal issues about administering those in the home”*
	*(P10): “The competencies, as you know, in general we will have the basic training but when it comes to the controlled drugs we need to know about the storage, dispensing, handling in the community setting, and waste disposal”*
	*(P3): “But I couldn’t stop thinking afterward about how that must have been just so emotional and upsetting that the family were under the impression that the medication had kind of hastened the death”*

b. Acceptance and community challenges

A substantial number of participants (*n* = 9) expressed their concerns about patient and family acceptance of the idea of PC and their understanding and expectations from the PC home program. They perceived family resistance as the main barrier in the community, especially when dealing with different family members with varying opinions. Language was another barrier to communication in the community for non-Arabic speaking staff. Some participants viewed treatment costs as a barrier for non-Qatari patients to access the service, as P6 explained: “The family, sometimes, is not accepting the idea of palliative care,” and P11 noted cost and language issues: “For expatriates, the cost might be a barrier, and language can make it hard to explain the service properly” (Supplementary Table 1).

c. Cultural and religious considerations

Another concern from most participants (*n* = 10) was related to cultural norms of what is and isn’t acceptable as well as the religious beliefs of the patient and the family that play a significant role in forming an agreeable PC plan of care. Qatar’s healthcare workforce is predominantly expatriate (comprising ∼88% of the population), with staff often originating from South Asia (e.g., India), the Philippines, and Arab countries, bringing diverse languages (primarily English and Arabic in professional use), religions (e.g., Hindu, Christian, Muslim), and cultural norms shaped by individualistic or varied family structures. In contrast, local Qatari patients and families, who are predominantly Muslim, adhere to conservative Islamic social norms emphasizing family-centered decision-making (often led by elders), spiritual preparation for death, and sensitivities around discussing end-of-life topics, which participants viewed as a barrier impacting the delivery of care and adherence to certain PC protocols, with P4 stating: “Cultural beliefs can affect acceptance of certain treatments, like discussing death openly,” and P7 adding: “Religious aspects must be respected, as they influence decisions on care plans” (Supplementary Table 2).

### 3.3 Communication and supportive services

a. Clear communication channels

Just over half of the participants (*n* = 7) highlighted the importance of having an agreed communication channel, through a named next of kin or legal guardian, especially when the patient lacks capacity, to ensure clarity in discussing PC decisions and avoid confusion or disagreements later, as P3 described: “We need a clear point of contact in the family to avoid mixed messages,” and P9 emphasized: “Having a designated family member helps in making timely decisions without conflicts” (Supplementary Table 3).

b. Psychosocial and spiritual support

A significant number of participants (*n* = 8) reflected on how PC is more than just about managing symptoms or controlling pain but also addressing the psychosocial and spiritual needs of both the patient and their families by availing psychological counseling as part of the service. Spiritual support is also needed, and one participant drew on their prior experience about the importance of providing chaplaincy service, illustrated by P2’s insight: “Spiritual care is crucial; in my experience, chaplaincy can provide comfort during end-of-life,” and P8 noting: “Psychosocial support helps families cope with the emotional burden” (Supplementary Table 4).

### 3.4 Training and resources

a. Staff training and emotional support

The majority of participants (*n* = 10) expressed the needs for training courses or workshops focusing on the appropriate skills for the delivery of PC at home. They also had concerns about their emotional health and wellbeing especially as they would often deal with EoL and dying patients, and to consider appropriate staff emotional support as part of the program, with P5 stating: “We need specialized workshops on palliative skills, including emotional resilience training,” and P10 adding: “Dealing with dying patients is tough; emotional support for staff is essential to prevent burnout” (Supplementary Table 5).

b. Policies and protocols

Effective PC need a multi-disciplinary approach, and many participants (*n* = 9) reflected on the importance of this to offer continuity of care, as well as having clear policies and protocols related to various aspects of PC care, especially the medico-legal issues surrounding consent, and administering medications at home, as P4 explained: “Clear protocols on consent and meds are needed for safe home administration,” and P7 highlighted: “A multi-disciplinary team ensures continuity, but we need policies to guide medico-legal aspects” (Supplementary Table 6).

c. Access to narcotic medications

All participants (*n* = 13) acknowledged the importance of timely access to narcotic medications to deliver effective pain management which is a key element in PC. However, they also voiced concerns about difficulties in accessing such medications, the injectable forms in particular, lack of protocols and pathways related to dispensing, storage in the home care hubs, transport, and administration. Furthermore, some thought it was something better left to be given in the hospital setting which meant the patient would have to be readmitted, especially when some families doubt its effect and view it negatively overall (Table 5).

## 4 Discussion

All participants had a good understanding of PC and EoL with the main focus on ensuring patients remain comfortable and pain-free to enhance their QoL through a patient-centered approach, which is consistent with the common consideration of PC delivery especially in the final year of life (16). Being pain-free is undeniably a high priority in PC, where a vision of “Freedom from cancer pain” was the catalyst for establishing the PC initiative in the regional Middle Eastern country Jordan (17). Respecting patients’ and families’ wishes was also an important element highlighted by the participants, and according to the same study, this would result in a better sense of support and comfort being provided at home to both patients and their families (16). Public awareness of PC was also frequently mentioned echoing the lack of education and the need to raise public awareness in Qatar to ensure meaningful progress in PC (6). On death and dying at home, most participants viewed dying at home in a familiar environment and among family members to be a peaceful death, away from the hospital, as long as this was consistent with the patient’s wishes. However, as pointed out earlier despite many patients wishing to die at home, the majority of cancer patients in Qatar still die in hospital (9). Similar findings were echoed in a Chinese study. Faced with the same burden by comparison, the authors stressed the need to establish palliative home care services in Macao. Despite the preferred place of death being a quality marker in PC, the paper acknowledged the disparity between the preferred and actual place of death (18).

On the theme of challenges and barriers, access and administration of narcotic drugs, the injectable forms in particular, were viewed to be the most significant. Lack of staff experience, safe storage, protocols, and legal issues were frequently mentioned concerns. In a study conducted in Qatar, the authors highlighted governmental laws and regulations restricting narcotic drug dispensing and use, coupled with a culture of fear both in terms of prescribing or causing patients to become addicted (11). The latest corporate policy in HMC also restricts the administration of narcotic drugs to in-patient or out-patient clinical settings, which means HHCS staff cannot administer those drugs at home which is an important issue that needs to be addressed on both corporate and ministerial levels to advance the PC program. Community acceptance, language barrier, and cost formed another sub-theme under challenges. Patients and families were often resistant according to the participants due to a lack of acceptance of PC. A Tanzanian study, “We never speak about death,” highlighted the need to have “a state of acceptance” through appropriate communication to provide PC (19). Similarly, in one Iranian study, 70% of patients didn’t accept to receive care at home resulting in patients being discharged from one hospital to another (20). Furthermore, while state healthcare is provided free of charge to all Qataris, and subsidized for expatriates (21), and with the introduction of mandatory healthcare insurance for expatriates (22), it remains unclear how this may impact their ability to access PC in the future. A third sub-theme was related to cultural and religious considerations, where participants expressed concerns regarding PC practices and protocols which may not align with the traditional norms and other cultural and religious beliefs. In this paper (12), the author acknowledged that while many Arab/Islamic countries were offering PC as an integral part of their healthcare systems, it was essential to establish culturally accepted guidance to inform its practices. An example of this can be found in this paper (5), where the authors cited possible social and religious factors as barriers to PC implementation, however, appreciating those cultural and religious aspects helped in shaping the DNR policy in Qatar and improving the acceptance of PC.

On the theme of communication and supportive services, participants expressed difficulty in establishing clear communication channels with the family especially when multiple family members are involved. In Qatari culture, it is often the eldest son who makes decisions when his elderly parent becomes sick (9), however, in practice, this is not always clear cut, especially in larger families where multiple members are involved which may confuse both the family and the healthcare team. Additionally, PC advance directive provisions have been instituted since 2004 in the state of Qatar (5), yet it isn’t commonly followed in practice. The reason for this is likely to do with having policies and practices that tend to be based on gold standard practice internationally, that aren’t locally nuanced or take into account local customs and traditions resulting in some form of disconnect which requires a culturally sensitive multi-sectoral approach to achieve success (12). Therefore, there is a need to ensure advance directive policies are implemented and communicated correctly to the family from the very beginning to ensure effective communication and timely decision-making for those involved. Another important insight highlighted the need to provide psychosocial and spiritual support to patients and their families. In PC, psychological aspects need appropriate consideration (23), and EoL requires different services not only addressing the physical but also the psychological and social needs both in the hospital and at home (16). The same was echoed in this study from Lebanon (24), therefore, under our PC plan, clinical psychologists will be recruited to offer this support. Considering the extent of the emotional burden on both PC patients and their families, Bharani et al. stressed the importance of providing a holistic approach including social support and religious chaplains in Qatar (5). The latter was mentioned by one of the participants (P2) and indeed is a vital element to consider during the PC service implementation.

The fourth and final theme was concerned with training and policies. Participants expressed the need for more PC focused training while at the same time providing them with emotional support. As alluded to earlier, only around a third of oncology physicians and nurses in Qatar had formal training in PC (7, 8). This reveals a significant gap that must be addressed to equip the staff with the required skills in various aspects of care (8). The need for tailored training while providing emotional support was evident in this study on the effect of the COVID-19 pandemic on PC nurses in Qatar to ensure resilience (25). Another concern was related to the lack of clear policies and protocols especially around consent and medication administration. Timely access to narcotic medications was identified as a priority in this study which must be supported with clear policies (21). In the context of Qatar, the WISH report was a step forward in proposing guidelines around palliative care locally (12). In their paper, Bharani et al. called upon policymakers to have clarity in terms of the availability and accessibility to essential drugs, develop PC standards, and have indicators in place to monitor their activity (5).

### 4.1 Limitations

Due to time constraints, the interview sessions coincided with the holy month of Ramadan when working hours are reduced. This may have impacted the number of participants or representation from other disciplines who would have otherwise opted to attend the interviews. Furthermore, the study did not include perspectives from underrepresented cadres such as allied health professionals (e.g., physiotherapists, occupational therapists) and social workers, whose insights might have provided additional depth on holistic care aspects, including psychosocial support, rehabilitation needs, and community integration in palliative care delivery. However, the 13 participants provided broad and diverse insights as shown under each theme and sub-theme enabling the study to produce relevant results and enriching the discussion about PC covering a multitude of perspectives, challenges, and opportunities.

Additionally, the study participants were exclusively selected from the HHCS, therefore their experiences and attitudes may not reflect the insights from clinical staff working in other departments and therefore may not be generalizable. However, the findings demonstrate notable parallels with hospital-based and regional studies from diverse contexts, such as those in Macao (18), Tanzania (19), Iran (20), and Lebanon (24), where similar challenges related to place of death preferences, community acceptance, cultural barriers, and communication issues were reported. This alignment suggests that key insights from this community-focused study may transfer to hospital settings or broader regional palliative care frameworks, particularly in culturally similar Middle Eastern and developing contexts. Furthermore, the voluntary participation may introduce selection bias, as individuals with greater interest, knowledge, or prior training in palliative care were more likely to respond, evidenced by the depth and informativeness of some comments in the results. We acknowledge that further studies which include participation from both hospital and community settings would be useful in highlighting other areas that may have not been identified in this study.

### 4.2 Implications for policy and practice

Since keeping patients’ pain-free and comfortable is a priority in PC, timely access to narcotic drugs becomes an essential goal fraught with challenges related to existing laws and policies which was acknowledged by all participants. In Qatar, receiving most narcotic medications is restricted to the inpatient or outpatient setting, with prescribing privileges limited to oncologists and 10 days of maximum supply (26). Furthermore, to reduce the pervasive culture of fear (11), a crucial reform to include a formulary and regulatory review is recommended to improve access to narcotic drugs in the community and to provide adequate training and education to healthcare staff to administer such medications at home.

Community resistance and acceptance issues can be addressed through targeted educational and awareness campaigns while considering the sensitivity of PC in the context of both cultural norms and religious beliefs and the need to develop culturally accepted guidelines (12).

Policies related to decision-making and advance directives must be followed to ensure clarity of communication and care planning, where directives become fundamental to life-limiting decisions (24). Psychosocial and spiritual issues are critical and must not be overlooked by focusing on pain and symptomatic management alone, therefore psychosocial support services are essential including the establishment of chaplaincy support (5).

On the HCP front, a tailored PC training program is vital in bridging knowledge and experience gaps among the home healthcare staff, while having clearly defined policies and protocols is recommended to ensure the delivery of care in its various aspects at the patient’s home (27).

## 5 Conclusion

Palliative and end-of-life care should be an integral part of any healthcare system, where the demand for such services will only increase due to aging trends globally. PC is relatively new in Qatar and initiatives to increase public awareness are needed. While many patients wish to die at home only very few do, resulting in an increased burden on hospitals. Having a home-based PC is key to delivering patient-centered care where the focus is on comfort and keeping patients pain-free. Patients’ and families’ wishes must be taken into consideration, especially on issues related to death and dying at home. This study highlights multiple barriers that must be addressed to ensure success in the delivery of PC service to patients cared for at home. Challenges to do with access to narcotic medications; laws and policies; culture and acceptance; education and awareness, were aligned with findings from other studies conducted both in Qatar and the Middle East region, all of which pose significant implications for practice.

The findings from this study provide a unique opportunity to understand the perceptions and attitudes of home healthcare staff toward PC in the state of Qatar, complementing the existing literature in the field which was primarily hospital focused. Furthermore, the study offers recommended approaches based on the highlighted challenges to a successful implementation of PC service in the HHCS and effective delivery of care in the home setting.

## Data Availability

The raw data supporting the conclusions of this article will be made available by the authors, without undue reservation.

## Appendix

Appendix 1 - Interview questions

Exploring perceptions and attitudes toward palliative care among healthcare professionals in Qatar’s home care setting

- What is your understanding of palliative care in the context of home healthcare?
  Prompts: end-of-life vs. palliative care.

- What are your personal beliefs and attitudes toward palliative care?
  Prompts: curative vs. symptomatic management, Death and dying at home.

- What kind of training and support do you feel are beneficial to palliative care staff in the homecare setting?
  Prompts: controlled drug administration.

- What are the main barriers to delivering effective palliative care in a home care environment?
  Prompts: cultural, religious, policies and protocols.
