# Correction: Perceptions and attitudes toward palliative care among healthcare professionals in Qatar's home care setting

**DOI:** 10.3389/fmed.2025.1728255

**Published:** 2025-11-12

**Authors:** Feras Haddad, Gary E. Day, Sybil George, Brijesh Sathian, Hanadi Al Hamad, Essa Al-Sulaiti

**Affiliations:** 1 Home Healthcare Service, Hamad Medical Corporation, Doha, Qatar; 2 ECA College of Health Sciences, Brisbane, QLD, Australia; 3 Department of Geriatrics and Long-Term Care, Rumailah Hospital, Doha, Qatar

**Keywords:** palliative care, end-of-life, Qatar, home healthcare, attitudes, barriers

Author Hanadi Al Hamad was erroneously spelled as Hanadi Al-Hamad.

There was a mistake in the caption of **Figure 3** as published. **Figure 3** is no longer a sunburst chart, but a code tree chart. The corrected caption of **Figure 3** appears below:

“Code tree chart: counts of codes and categories.”

The **Conflict of interest** statement was erroneously given as FH, SG, and EA-S were employed by Hamad Medical Corporation. The remaining authors declare that the research was conducted in the absence of any commercial or financial relationships that could be construed as a potential conflict of interest.

The correct **Conflict of interest** statement is:

FH, SG, BS, HA and EA-S were employed by Hamad Medical Corporation.

The remaining author declares that the research was conducted in the absence of any commercial or financial relationships that could be construed as a potential conflict of interest.

The following text has been removed from: **1 Introduction**, Paragraph Number 03

The multidisciplinary team includes physicians, nurses, occupational therapists, dieticians, respiratory therapists, and support staff, trained through HMC's accredited programs emphasizing clinical skills, patient safety, and person-centered care aligned with JCI standards. Patients are referred primarily by PHCC doctors or private clinics, with online submissions via hamad.qa for non-PHCC referrals requiring health card details and forms, followed by scheduling through customer service. Services encompass clinical assessments, symptom management, functional training, home modifications, fall prevention, and education for patients and caregivers on self-care and equipment use. Visits are tailored to patient needs, occurring regularly for chronic cases or as needed for interventions, with over 22,300 annual home visits supporting reduced hospital admissions.

The original version of this article has been updated.

