# Correction: Perceptions and attitudes toward palliative care among healthcare professionals in Qatar's home care setting

**DOI:** 10.3389/fmed.2025.1764325

**Published:** 2026-01-12

**Authors:** Feras Haddad, Gary E. Day, Sybil George, Brijesh Sathian, Hanadi Al Hamad, Essa Al-Sulaiti

**Affiliations:** 1 Home Healthcare Service, Hamad Medical Corporation, Doha, Qatar; 2 ECA College of Health Sciences, Brisbane, QLD, Australia; 3 Department of Geriatrics and Long-Term Care, Rumailah Hospital, Doha, Qatar

**Keywords:** palliative care, end-of-life, Qatar, home healthcare, attitudes, barriers

The Figures 1, 2 and the captions were in the wrong order in the PDF/HTML version of this paper.

The corrected Figures 1, 2 and its caption appears below.

**Figure 1:**
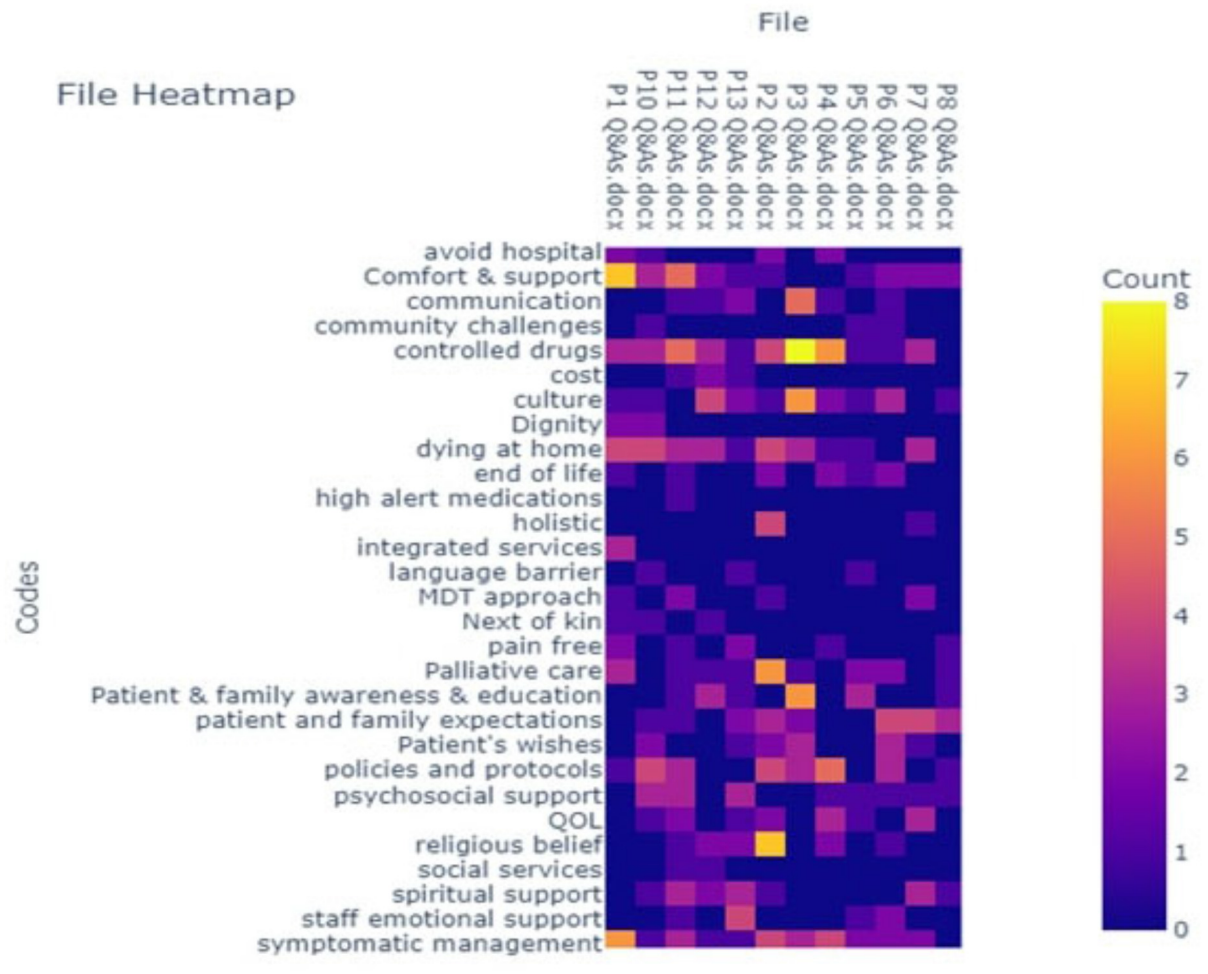
Codes heatmap.

**Figure 2:**
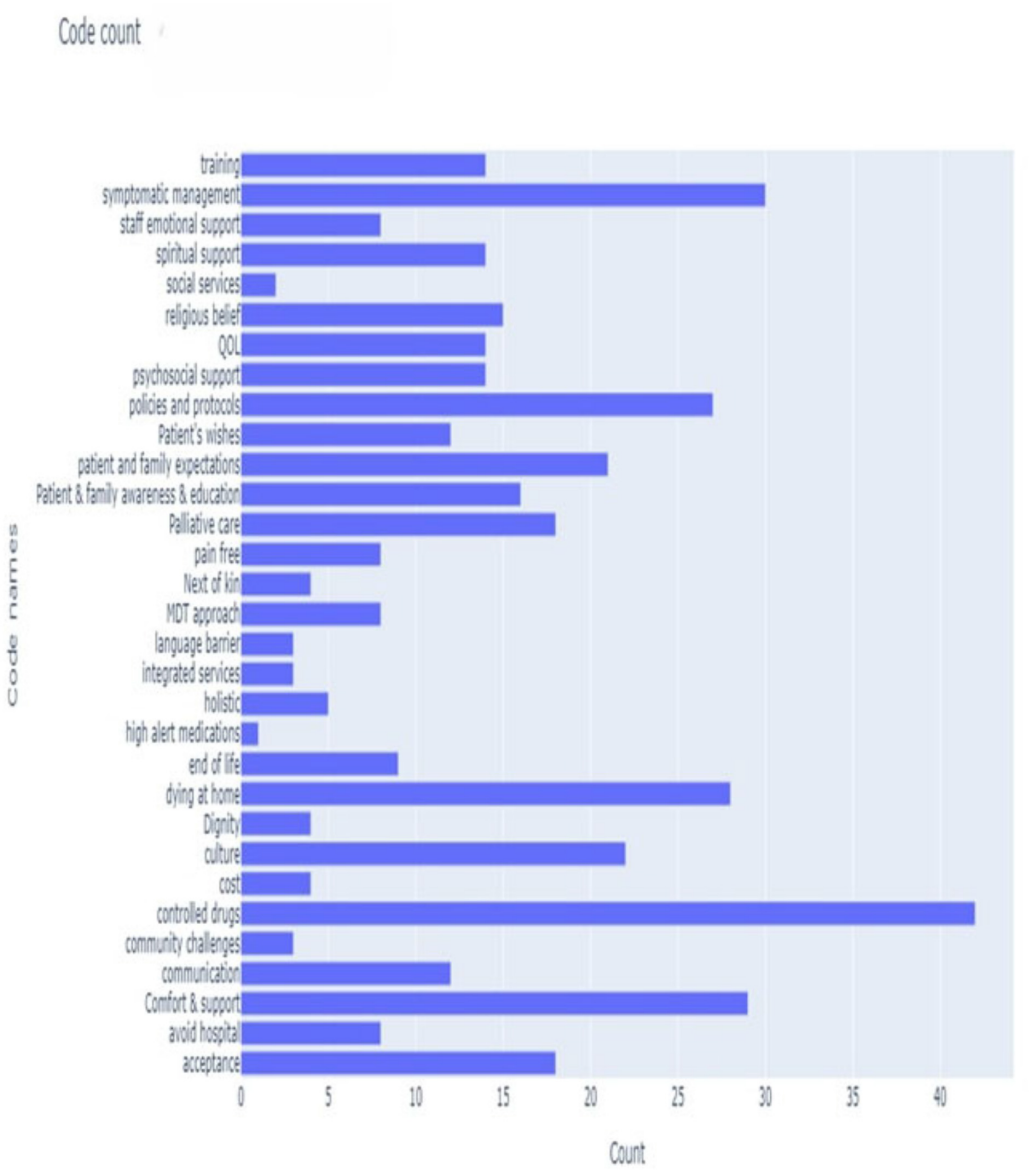
Bar chart: code count.

The original version of this article has been updated.

