# Supplementary material for: Perceptions and attitudes toward palliative care among healthcare professionals in Qatar’s home care setting
Source: Front Med (Lausanne). 2025 Oct 9;12:1678462. doi: 10.3389/fmed.2025.1678462 (PMC12546369; doi:10.3389/fmed.2025.1678462)
Supplement: Supplementary file 1 [file Data_Sheet_1.docx]

**LEGEND**

Supplementary Table ST1 - quotes: acceptance and community challenges

Supplementary Table ST2 - quotes: cultural and religious considerations

Supplementary Table ST3 - quotes: clear communication channels

Supplementary Table St4 - quotes: psychosocial and spiritual support

Supplementary Table St5 - quotes: staff training and emotional support

Supplementary Table St6 - quotes: policies and protocols

Supplementary Table ST1 - quotes: acceptance and community challenges

| **Sub-theme** | **Quotes** |
| --- | --- |
| **Acceptance and community challenges** | *(P2): “The lack of insight and lack of patient and family acceptance of the bad prognosis, that's the main barrier”*  *(P4): “Some may believe it's not ethical, I mean I'm talking about the families, so they may find it very hard to withdraw care or to stop medications, blood sugar medications or any other medicines for a chronic disease and leaving him to die”*  *(P3): “I discussed it with different family members. He had no idea of what was going on and what had been discussed in the morning. He did not agree with the DNR”*  *(P6): “I think a major thing would be a conflict between what palliative care can offer and what the patient and family expect”*  *(P5): “As a non-Arabic person, I really find it sometimes difficult to converse with or console the family members”*  *(P11): “In my perspective, I don't know regarding non-Qatari patients, maybe they will get the same free service. But the high cost may be one problem impacting the acceptance of the family members”* |

Supplementary Table ST2 - quotes: cultural and religious considerations

| **Sub-theme** | **Quotes** |
| --- | --- |
|  |  |
| **Cultural and religious considerations** | *(P10): “Palliative care is a very sensitive thing. We need to understand the cultural differences to have effective palliative care at home setting”*  *(P12): “The local traditions, you know. It's very difficult when you are at home and if you know someone is dying and how to communicate this”, “Many cultural beliefs of the patient’s family can affect the patient care” (P6): “I think the cultural beliefs or religious beliefs, because sometimes as palliative care clinical staff, we have certain protocols. We have certain dos and don’ts which don’t always match with what the family or the patient are demanding”*  *(P2)” There is a lot of literature that says patients with religious belief deal with death and dying much differently to patients without much of a religious inclined belief”*  *(P4): “They feel that we have to treat to the last extent of the available medical technology or medical knowledge. Some patients look at this from a religious point of view and this is why you can find resistance in some family members and sometimes denial”* |

Supplementary Table ST3 - quotes: clear communication channels

| **Sub-theme** | **Quotes** |
| --- | --- |
| **Clear communication channels** | *(P12): “I mean the communication channel, whom we can call or contact, who should have a proper awareness of this situation” (P3): “Just the sheer number of people you will have to talk to again to get a family consensus especially obviously where the patient is really in the end of life and they can't communicate, you know that will be something challenging” (P1): “If the patient obviously is unconscious or demented, or can't make a decision, then it is the next of kin who will make that decision”* |

Supplementary Table St4 - quotes: psychosocial and spiritual support

| **Sub-theme** | **Quotes** |
| --- | --- |
| **Psychosocial and spiritual support** | *(P10): “Keeping in mind the person. I mean the patient and the families as a whole to consider their all needs, not only those physical symptoms, but the other needs of the patient like social or spiritual needs as well” (P12): “We don't have as you know any service to meet the spiritual needs in home care” (P2): “So you will have to take all of these things into consideration and offer psychological support chaplaincy support to patients”, “it should be offered to patients who are dying at home, religious support, chaplaincy service should be offered. It’s crucial”* |

Supplementary Table St5 - quotes: staff training and emotional support

| **Sub-theme** | **Quotes** |
| --- | --- |
| **Staff training and emotional support** | *(P13):” I think we have to be emotionally ready to empathize with the family, to understand that the patient and the family are already suffering, so we need to be psychologically fit, mentally fit at the same time.” (P5): “We are dealing with these cases and sometimes our patients die suddenly, or something happens. We are even affected on certain days” (P6):” We need a workshop for this kind of scenario to be explained to the staff. I think they could be able to handle situations better”  (P2): “We should have practical clinical exposure to that before we can independently look after these patients out in the Community”  (P10): “So we need to enrol the palliative care staff in a program or specific training pertaining to that because in the community setting it will be very different than the hospital”* |

Supplementary Table St6 - quotes: policies and protocols

| **Sub-theme** | **Quotes** |
| --- | --- |
| **Policies and protocols** | *(P1): “It's a multi-disciplinary team that needs to sit down together and go through the case” (P2): “The allied health starting from nursing staff, respiratory therapists, physical therapists, occupational therapists, SLP, or clinical pharmacist. All of them would essentially need exposure to the terminal stages of life” (P10): “What are the policies and procedures that we have in the hospital and also the department if we wish to give any drugs at home? I am not very sure about this”  (P2): “And then we need a clear pathway for the medico-legal stuff. Medical law or Mental Health Act for patients’ autonomy and informed consent” (P4): “If the patient was not able to have it through an oral route we need to find another route of administration. We cannot do that because of the medico-legal issues”* |
